# Using evolutionary theory to enhance the brain imaging paradigm

**DOI:** 10.3389/fnhum.2014.00452

**Published:** 2014-06-20

**Authors:** Gad Saad, Gil Greengross

**Affiliations:** Marketing Department, John Molson School of Business, Concordia UniversityMontreal, QC, Canada

**Keywords:** neuroimaging, evolutionary psychology, illusion of explanatory depth, scientific method, ecological validity, domain-specific modules, proximate vs. ultimate

In recent years, there seems to be no preference, choice, emotion, thought, or behavior that has escaped the scrutiny of a neuroimaging machine. Scanning the brain allegedly reveals insights into the foundations of morality (Greene et al., 2001), altruism (Tankersley et al., 2007), sense of humor (Bartolo et al., 2006) and even religious beliefs and God (Kapogiannis et al., 2009), to name just a few of the disparate topics that have been studied. As neuroimaging studies become increasingly popular, a growing number of researchers in the business disciplines are applying such techniques within their areas of interest. In such works, researchers look at and map the parts of the brain that are involved in processing decisions, preferences, and choices. Studies range from predicting future sales of popular songs based on how certain areas in the brain were activated on a sample of individuals prior to a song's release (Berns and Moore, 2012), how advertisements using various forms of persuasion engage different parts of the brain (Cook et al., 2011), and how arbitrary prices of wine alter the reward activities in an area of the brain associated with pleasure (Plassmann et al., 2008).

The modus operandi of most imaging procedures, such as fMRI, is to track blood oxygenation in the brain with the underlying assumption that as blood flows to a region, the more neurons are activated in this area. The idea behind such studies is not only to locate the exact area in the brain where information is processed, but also to have insights into people's true thoughts and preferences, since individuals cannot control their brain activity and are not always consciously aware of their thought processes. There is a prevailing view that by looking at individuals' brain activation patterns, we could unveil their latent desires. Ariely and Berns (2010) discuss this premise skeptically, specifically in the marketing discipline, where there is a nagging fear that by looking at people's brains, marketers would be able to predict individuals' penchants for certain products, future acquisitions and needs, and hence manipulate customers to take advantage of those desires.

This fear is probably unwarranted and stems from what Rozenblit and Keil (2002) describe as “the illusion of explanatory depth,” namely to exhibit overconfidence in one's ability to offer veridical explanations about natural phenomena when one's true knowledge is tentative at best. Neuroimaging scholars are especially susceptible to such biases. The striking colorful brain photos and associated technical jargon have a persuasive effect on researchers and lay person alike (McCabe and Castel, 2008; Trout, 2008; Weisberg et al., 2008), but this should not blind us to some of the shortcomings of the paradigm including the likelihood of reporting spurious correlations (Vul et al., 2009) and false positives such as the infamous case of the neuronal activation patterns “found” in a dead Atlantic salmon (Bennett et al., 2010). Next we detail some of the problems in neuroimaging research that need to be taken into account, and offer a theoretical framework that might be helpful in better interpreting the reaped results.

There are several practical problems associated with the neuroimaging paradigm. Imaging studies are typically conducted in artificial settings where subjects are physically constrained within a narrow and claustrophobic device which may lead to a selection bias. The contrived laboratory environments lack ecological validity and as such it is unclear whether the neuronal activation patterns would be the same were participants making real and consequential choices. To make an actual moral choice in real life differs from having to imagine making a hypothetical one whilst lying down in a machine. Brain scans are also quite costly and thus sample sizes are typically quite small, yielding low statistical power, a possible overestimation of effects sizes, or the failure to detect true effects when they indeed exist (Button et al., 2013). Underpowered studies are hard to replicate and they often lead to selection biases in published results. Such studies can either report bogus results or can lead to the file drawer effect (e.g., unpublished positive results that did not reach significant levels due to small samples). These problems are relatively easy to fix with more rigorous study designs that include adequate sample sizes and transparency regarding power calculations.

A more fundamental problem in neuroimaging studies is the inability to identify the exact area in the brain responsible for a given cognitive process. The brain, which consumes roughly 20% of all energy in our body, is responsible for monitoring and managing every human activity, and is built in a complex network where different modules work simultaneously for almost any task (see Pinker, 1997). At any moment, multiple areas are activated concurrently, and it is not easy to pinpoint the one region that is responsible for a certain thought or desire, if one such place even exists. Neuroimaging studies are adept at illuminating areas in the brain that are associated with certain behaviors, thoughts, or preferences, but interpreting which functions these areas serve based on the images is difficult and typically cannot be derived directly from such images. Moreover, highlighted areas in the brain do not exclude the possibility that other parts of it are also involved, as these parts may already be activated but not show additional activity with the new task (Lee et al., 2012). That said, recent studies have documented the ability to classify mental states, namely to accurately map a mental task with a particular activation pattern (Poldrack et al., 2009).

Locating a neuronal activation pattern in the brain tells us little about the underlying causes that led to the cortical activity in question. To better understand the causal mechanisms that lead individuals to act the way they do, we need a meta-theory one that has the power to explain the ultimate causes of behaviors and preferences and not just help “locate” them in the brain. Evolution is the only scientific theory that could explain the underlying ultimate causes of behaviors and preferences, and the forces that shaped them via natural and sexual selection processes. The ultimate goal of every organism is to survive and reproduce and thus, inquiries into the functionality of the human brain require the evolutionary lens. That said, the great majority of neuroimaging studies fall within the proximate realm (address *how* and *what* factors), and as such they seldom seek to elucidate the Darwinian genesis of neuronal processes (ultimate causation). It is one thing to detail where in the brain emotions such as fear, love, or anger “reside,” but another epistemological lens is needed to understand why humans possess the ability to fear or to love, under what circumstances these emotions are activated, and which evolutionary purpose they serve in terms of an individual's survival and reproduction outlooks. It is crucial to differentiate between how cognitive processes manifest themselves in the brain at the neural level and the evolutionary pressures leading to the existence of such structures. The identification of neural activities is important and can shed some light on their purpose, but without recognizing specific evolutionary mechanisms such as natural, sexual, and kin selection, we cannot fully infer why they came to be in the first place (see Senior et al., 2011).

A first step toward Darwinizing the brain imaging paradigm would be to make greater use of evolutionarily meaningful stimuli and tasks (photos of a juicy burger or a sexy prospective mate) instead of largely relying on abstract domain-general stimuli and tasks (playing chess or choosing between probabilistic gambles). Moreover, using an evolutionary framework can help generate context-specific stimuli that are differentially relevant to various demographic groups. For example, if we wish to examine how sexual arousal is expressed in the brain, knowing the evolutionary roots of sexual fantasies and how men and women differ in their responses to visual stimuli (Ellis and Symons, 1990) can help in devising experimental tasks that would produce sex-specific arousal (e.g., explicit vs. non-explicit photos). More generally, evolutionary thinking can contribute to neuroimaging research by invoking domain-specific processes that map onto key basal Darwinian modules including survival, mating, kin selection, and reciprocity (Platek et al., 2007; Saad, 2007, 2011).

The examination of the four Darwinian modules has yielded new insights when applied to neuroimaging research. In a study pertaining to the survival module, the mere exposure to pictures of highly caloric food produced brain activation in areas associated with taste and rewards, similar to ones that are triggered in response to real food (Simmons et al., 2005). Of relevance to the mating module, researchers found that when faces of attractive women are presented to men, these activate reward systems in the brain that had previously been identified as responsible for other powerful rewards such as drugs and money (Aharon et al., 2001). Using kin selection principles, Platek and Kemp (2009) showed how different parts of the medial substrates in the brain are activated in response to faces of kin, non-kin friends, and strangers. This makes evolutionary sense since facial categorization and the ability to distinguish between kin and non-kin have important consequences for survival (differentiating a friend from a foe) and reproduction (avoiding incest with a family member). More generally, the ability to recognize human faces is itself adaptive. Using an evolutionary perspective, researchers have shown that the medial frontal cortex was much more activated when making a decision about whether to trust another person, but not when interacting with an avatar (Riedl et al., 2014). Lastly, various works have explored neural processes associated with the reciprocity module including identifying specific areas in the brain that are activated during moral dilemmas that require cooperation (Singer et al., 2004) and detecting cheaters (Stone et al., 2002).

Ultimately, the exploration of evolved domain-specific modules (rather than domain-general cognitive processes) via the use of ecologically relevant stimuli and tasks will yield a consilient brain atlas. Furthermore, it will likely reduce the “fishing expedition for statistical significance” feel of many neuroimaging studies, by permitting for more ecologically relevant study designs and by facilitating the positing of a priori hypotheses.

Given the apparent methodological sophistication of the brain imaging paradigm, neuroscientists are particularly prone to what the Darwinian philosopher Daniel Dennett referred to as “greedy reductionism” (Dennett, 1995). Endless studies are conducted void of any organizing theory or guiding a priori hypotheses. Rather, the sophisticated methodology drives the epistemological engine. In a survey of 50 neuroimaging studies only 42% (21 papers) included *a priori* hypotheses (Garcia and Saad, 2008). Of these, 15 were evolutionary based and 6 were non-evolutionary based. Most striking is the fact that only 17 of the 50 papers took an evolutionary approach in the first place, meaning that 88.2% of the evolutionary papers posited *a priori* hypotheses, where only 18.2% of the non-evolutionary papers used such hypotheses. In other words, an evolutionary framework is much more likely to generate *a priori* hypotheses that can be tested using imaging devices, where non-evolutionary approaches produce many more *ad-hoc* and *post-hoc* explanations. Thus, a parsimonious and integrative framework such as evolutionary theory serves as a safeguard of the scientific method.

While some have described the neuroimaging paradigm as the new phrenology (Uttal, 2001), we do not share such a pessimistic outlook. Neuroscience is still a nascent and rapidly developing field, and some of the criticism is overstated (Farah, 2014). Recently, the United States and the European Union announced two ambitious projects: the BRAIN Initiative and the Human Brain Project. Key objectives include mapping the brain as well as creating a full simulation of it, which could not only help us in better understanding the human mind but also could help in combatting various brain and mental illnesses. Similar to previously innovative technologies such as the telescope and the microscope, brain imaging machines are merely tools that need to be used within a specific meta-theory. The evolutionary framework could provide a good starting point as an overarching theory to better organize and fully understand the ultimate mechanisms that drive our behaviors, emotions, and thoughts, as seen in such lively brain images.

## Conflict of interest statement

The authors declare that the research was conducted in the absence of any commercial or financial relationships that could be construed as a potential conflict of interest.
